# Increased killing of SCCVII squamous cell carcinoma cells after the combination of Pc 4 photodynamic therapy and dasatinib is associated with enhanced caspase-3 activity and ceramide synthase 1 upregulation

**DOI:** 10.3892/ijo.2013.2132

**Published:** 2013-10-09

**Authors:** DUSKA SEPAROVIC, PAUL BREEN, NITHIN B. BOPPANA, ERIC VAN BUREN, NICHOLAS JOSEPH, JACQUELINE M. KRAVEKA, MEHRDAD RAHMANIYAN, LI LI, TATYANA I. GUDZ, ALICJA BIELAWSKA, AIPING BAI, JACEK BIELAWSKI, JASON S. PIERCE, MLADEN KORBELIK

**Affiliations:** 1 Department of Pharmaceutical Sciences, Eugene Applebaum College of Pharmacy and Health Sciences, Wayne State University, Detroit, MI 48201;; 2 Karmanos Cancer Institute, Wayne State University, Detroit, MI 48201;; 3 Department of Pediatrics, Division of Hematology-Oncology, Charles Darby Children’s Research Institute, and Hollings Cancer Center, Medical University of South Carolina, Charleston, SC 29425, USA,; 4 Ralph H. Johnson Veterans Affairs Medical Center, and Department of Neuroscience, Medical University of South Carolina, Charleston, SC 29425, USA;; 5 Department of Biochemistry and Molecular Biology, Medical University of South Carolina, Charleston, SC 29425, USA;; 6 British Columbia Cancer Agency, Vancouver, BC V5Z 1L3, Canada

**Keywords:** apoptosis, ceramide, ceramide synthase, ceramidase, dasatinib, PDT, sphingolipids, sphingosine

## Abstract

Photodynamic therapy (PDT) is not always effective as an anticancer treatment, therefore, PDT is combined with other anticancer agents for improved efficacy. The combination of dasatinib and PDT with the silicone phthalocyanine photosensitizer Pc 4 was assessed for increased killing of SCCVII mouse squamous cell carcinoma cells, a preclinical model of head and neck squamous cell carcinoma, using apoptotic markers and colony formation as experimental end-points. Because each of these treatments regulates the metabolism of the sphingolipid ceramide, their effects on mRNA levels of ceramide synthase, a ceramide-producing enzyme, and the sphingolipid profile were determined. PDT + dasatinib induced an additive loss of clonogenicity. Unlike PDT alone or PDT + dasatinib, dasatinib induced zVAD-fmk-dependent cell killing. PDT or dasatinib-induced caspase-3 activation was potentiated after the combination. PDT alone induced mitochondrial depolarization, and the effect was inhibited after the combination. Annexin V^+^ and propidium iodide+ cells remained at control levels after treatments. In contrast to PDT alone, dasatinib induced upregulation of ceramide synthase 1 mRNA, and the effect was enhanced after the combination. Dasatinib induced a modest increase in C20:1-and C22-ceramide but had no effect on total ceramide levels. PDT increased the levels of 12 individual ceramides and total ceramides, and the addition of dasatinib did not affect these increases. PDT alone decreased substantially sphingosine levels and inhibited the activity of acid ceramidase, an enzyme that converts ceramide to sphingosine. The data suggest that PDT-induced increases in ceramide levels do not correlate with ceramide synthase mRNA levels but rather with inhibition of ceramidase. Cell killing was zVAD-fmk-sensitive after dasatinib but not after either PDT or the combination and enhanced cell killing after the combination correlated with potentiated caspase-3 activation and upregulation of ceramide synthase 1 mRNA but not the production of ceramide. The data imply potential significance of the combination for cancer treatment.

## Introduction

Photodynamic therapy (PDT), a non-invasive cancer treatment modality, can effectively eradicate local malignancies. PDT utilizes a light-absorbing photosensitizer, visible light and oxygen to generate reactive oxygen species that destroy malignant cellular targets (1). However, because tumors recur, PDT needs to be optimized to improve its therapeutic benefit. Dasatinib, a multi-kinase inhibitor, is an anticancer agent that has been successfully used for treatment of chronic myeloid leukemia (2). As a single agent evaluated in clinical trials for treatment of solid tumors, including advanced head and neck squamous cell carcinoma (HNSCC), however, dasatinib has not been shown to be successful (3). In combination with other chemotherapeutic agents or radiation dasatinib is a more effective anticancer treatment *in vitro*, *in vivo* and in clinical trials (4–7).

**Figure 1.**
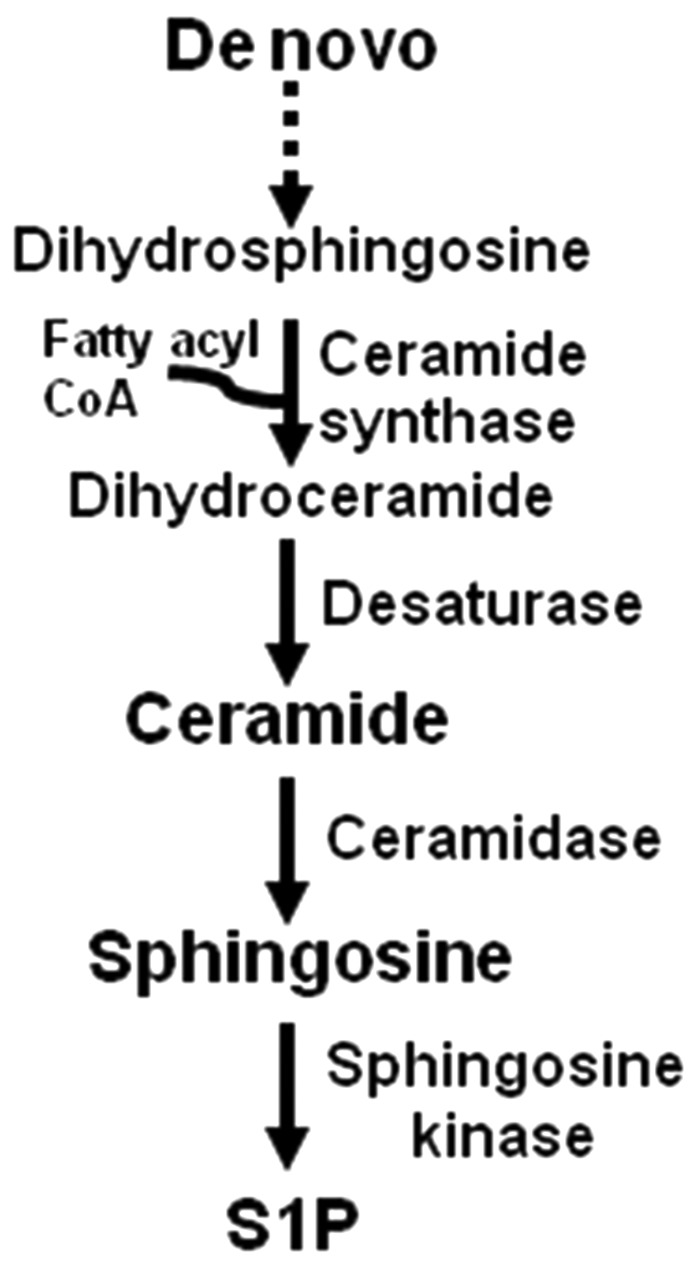
Ceramide metabolism.

Bioactive sphingolipids have been implicated in drug-and radiation-resistance, therefore targeting sphingolipid metabolism can contribute to increased effectiveness of the current treatment strategies (8). As shown in Fig. 1, the sphingolipid ceramide is generated in the *de novo* biosynthesis pathway, which includes a ceramide synthase-dependent addition of a fatty acyl group to dihydrosphingosine to form dihydroceramide. Ceramide is formed from dihydroceramide by a desaturase-dependent insertion of a double bond in the sphingosine backbone. Six mammalian ceramide synthases have been identified with distinct specificity for fatty acyl CoAs and functions (9). For example, C18- and C16-ceramide, containing an 18- and 16-carbon fatty acid, are generated by ceramide synthase 1 and 6, respectively, and induce HNSCC suppression and proliferation, respectively (10). Ceramide is deacylated by ceramidase, giving rise to sphingosine, and sphingosine is acted upon by sphingosine kinase to give rise to sphingosine-1-phosphate (S1P), an antiapoptotic sphingolipid.

We demonstrated that the knockdown of ceramide synthase 1 or 6 is associated with reduction in ceramides and dihydroceramides resulting in apoptotic resistance to PDT with Pc 4 (11,12). Dasatinib induces apoptosis via upregulation of ceramide synthases, including increased expression of *ceramide synthase 1* gene (13). The combination of dasatinib and PDT with Pc 4 was tested for potential anticancer efficacy in SCCVII mouse squamous cell carcinoma cells, a preclinical model of HNSCC (14), using apoptotic markers, colony formation and ceramide metabolism as experimental end-points.

## Materials and methods

### Materials

The phthalocyanine photosensitizer Pc 4, HOSiPcOSi(CH3)_2_(CH2)_3_N(CH_3_)_2_, was supplied by Dr Malcolm E. Kenney (Department of Chemistry, Case Western Reserve University, Cleveland, OH, USA). N-[9,10-^3^H]D-e-C16-ceramide was synthesized at the Lipidomics Shared Resource (Medical University of South Carolina, Charleston, SC, USA). RPMI medium and serum were from Life Technologies (Carlsbad, CA, USA) and Hyclone (Logan, UT, USA), respectively. The inhibitors zVAD-fmk and dasatinib (BMS-354825) were from MBL International (Woburn, MA, USA) and Selleck Chemicals (Houston, TX, USA), respectively.

### Cell culture and treatments

SCCVII cells, initially derived from the spontaneous abdominal wall tumor of a C3H mouse (15), were grown in RPMI medium containing 10% fetal bovine serum, 100 U/ml penicillin and 100 *μ*g/ml streptomycin (Life Technologies). Cells were maintained at 37°C in a 5% CO_2_ atmosphere and were treated in the growth medium. For PDT experiments, after overnight incubation with Pc 4 at 37°C, cells were irradiated with red light (2 mW/cm^2^; λ_max_ ∼670 nm) using a light-emitting diode array light source (EFOS, Mississauga, ON, Canada) at the fluence of 200 mJ/cm^2^ at room temperature and then incubated at 37°C for indicated periods of time. For PDT + dasatinib, dasatinib was added to the cells 22 h prior to irradiation, unless indicated otherwise. After treatments, cells were collected on ice and processed for various analyses. For mass spectroscopy (MS) analysis, cells were washed twice with cold phosphate-buffered saline (Corning Life Sciences, New York, NY, USA), resuspended in the mixture of ethyl acetate/methanol (1:1, v/v; EMD Chemicals, Billercia, MA, USA), dried under nitrogen and shipped overnight on dry ice to the Lipidomics Shared Resource (Medical University of South Carolina, SC, USA) for further processing.

### Electrospray ionization/double mass spectrometry (MS) analysis

After extraction, sphingolipids were separated by high performance liquid chromatography, introduced to electrospray ionization source and then analyzed by double MS using TSQ Quantum Access Max triple stage quadrupole mass spectrometer (Thermo-Fisher Scientific, Pittsburg, PA, USA) as described previously (16).

### RNA extraction and quantitative real-time polymerase chain reaction (RT-PCR)

Total RNA isolation was performed with RNeasy^®^ Mini kit (Qiagen, Valencia, CA, USA) according to the manufacturer’s instructions. cDNA was synthesized from 1 *μ*g of the total RNA using iScript™ cDNA Synthesis kit (Bio-Rad, Hercules, CA, USA). The concentration and quality of total RNA preparations were evaluated spectrophotometrically. RT-PCR was performed on a Bio-Rad CFX96 detection system using Bio-Rad SsoFast Probes Supermix™ and TaqMan^®^ Gene Expression Assays (Life Technologies) with the primers for ceramide synthases 1, 2, 4, 5, 6, the housekeeping gene products RPL37A and hypoxanthine-guanine phosphoribosyltransferase (HGPRT), and the fluorophore probe FAM-490 (6-carboxyflurescein; all obtained from Life Technologies). Initial steps of RT-PCR were 30 sec at 85°C, followed by 40 cycles consisting of a 5 sec at 95°C, followed by 10 sec at 60°C. Determination of the relative normalized expression of corresponding ceramide synthase mRNAs against the expression of housekeeping gene-encoded proteins RPL37A and HGPRT was performed by ΔΔC_T_ provided by CFX96 manager software 3.0 from Bio-Rad.

### Acid ceramidase activity assay

Acid ceramidase activity was performed as described previously (17). Cells were lysed under acidic condition (pH 4.5). Equal amounts of N-[9,10-^3^H] D-e-C16-ceramide were mixed with 0.2% Triton X-100 and 0.4% cholate and dried down under nitrogen. The lipid film was dissolved by mixing and sonication in deionized water. After additions of acidic assay buffer (0.2 M acetic acid, 0.2 M sodium acetate and 0.5% Triton X-100, pH 4.5) and cell lysate, the reaction was carried at 37°C for 1 h and stopped by adding Dole’s alkaline solution. [^3^H]palmitic acid, a hydrolytic product of acid ceramidase, was extracted and processed to calculate the enzyme activity (17). Quantitation of radioactivity was performed using LS 6500 multipurpose scintillation counter (Beckman Coulter, Brea, CA, USA).

### DEVDase (caspase-3) activity assay

As described previously (18), DEVDase activity was determined in the cytosol by an assay based on the enzyme’s cleavage of a fluorogenic derivative of the tetrapeptide substrate N-acetyl-Asp-Glu-Val-Asp (DEVD; Enzo Life Sciences, Farmingdale, NY, USA). The peptide sequence is based on the cleavage site Asp^216^ of the caspase-3 substrate poly(ADP-ribose) polymerase (PARP). The fluorescence of the cleaved DEVD substrate was measured using a spectrofluorometer (F-2500 Hitachi, New York, NY, USA; 380 nm excitation, 460 nm emission).

### Mitochondrial depolarization measurement

The lipophilic cationic dye JC-1 (5,5′,6,6′-tetrachloro-1,1′3,3′-tetraethylbenzimidazolylcarbocyanine iodide; BD Biosciences, San Diego, CA, USA) was used to determine mitochondrial membrane potential by flow cytometry, as we described previously (11,19,20). After treatments, cells were harvested and processed for flow cytometry according to the manufacturer’s instructions (BD Biosciences). BD LSR II flow cytometer was used for analysis (BD Biosciences).

### Apoptosis detection

As previously described (11,20,21), to detect apoptosis, the exposure of phosphatidylserine in the outer leaflet of the cell membrane and cell membrane integrity loss were measured using Annexin V and DNA-binding propidium iodide fluorescent dyes (BD Biosciences), respectively. Early apoptotic (Annexin V^+^/propidium iodide^−^) were distinguished from late apoptotic or necrotic cells (Annexin V^+^/propidium iodide^+^). The kit was obtained from BD Biosciences and the flow cytometric protocol was followed, as described by the manufacturer.

### Clonogenic assay

Long-term cell viability was assessed using clonogenic assay according to the modified protocol that we described previously (20). Plating density was 250 cells/plate. Plating efficiency was 34% (n=16).

### Protein determination

Protein content was determined by a modified Bradford assay (Bio-Rad) or, for acid ceramidase assay, by a bicinchoninic acid protein assay kit (Thermo-Fisher Scientific).

### Statistical analysis

Data are shown as the mean ± SEM. Statistical analyses were performed by Student’s t-test. Significance was defined as a two-tailed p<0.05.

## Results

### PDT + dasatinib enhances overall cell killing. Effect of zVAD-fmk on PDT ± dasatinib-induced cell killing

**Figure 2.**
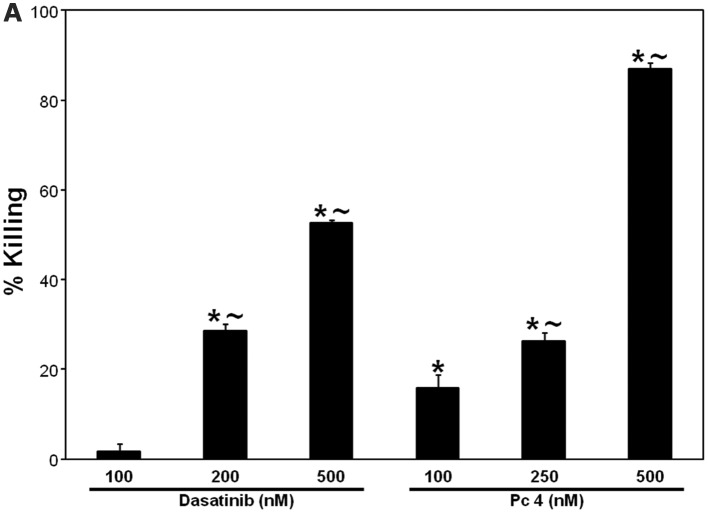

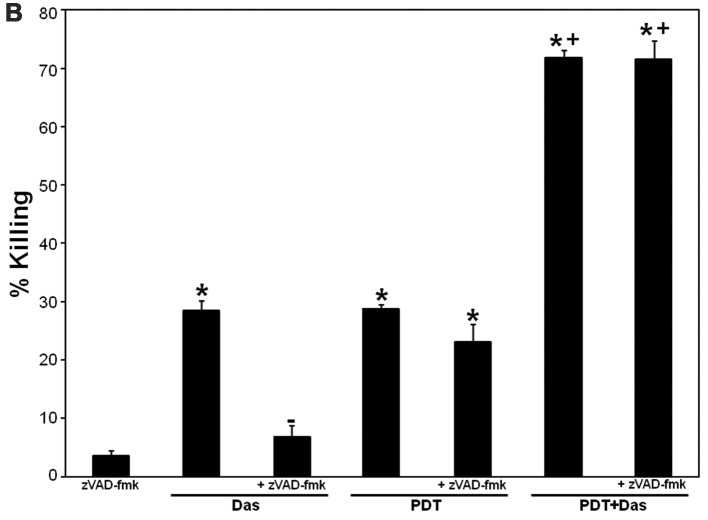
(A) PDT and dasatinib, respectively, induce dose-dependent cell killing. (B) Effect of zVAD-fmk on PDT ± dasatinib-induced cell killing. SCCVII cells were plated after appropriate dilutions into P60-mm dishes and allowed to attach overnight in the growth medium. For PDT, cells were plated in the growth medium containing Pc 4 at the indicated concentrations (A) or at 250 nM (B), incubated overnight at 37°C, and irradiated with red light (200 mJ/cm^2^). (B) zVAD-fmk (25 *μ*M), was added 1 h prior to treatments; For PDT + dasatinib, dasatinib (200 nM) was added immediately prior to irradiation. (A and B) After 8–10 days of growth at 37°C, colonies (≥50 cells) were stained with crystal violet (0.1%) and counted. The data are expressed as the percentage of killing and are shown as the mean ± SEM, n=3–12. The significance (p<0.05) is shown as follows: ^∼^difference between corresponding doses of dasatinib or PDT; ^*^treated are different from untreated control; ^+^(PDT + dasatinib) is different from PDT or dasatinib alone; ^−^zVAD-fmk is different from dasatinib alone. Das, dasatinib.

To test whether killing of SCCVII cells is increased by the combination of Pc 4-PDT with dasatinib, colony formation assay was used as the experimental end-point. The treatments were first used as single agents to determine whether they induce dose-dependent cell killing. Incubation of SCCVII cells with 100, 200 and 500 nM dasatinib led to 2, 28 and 53% cell killing, respectively (Fig. 2A). Similarly, PDT with 100, 250 or 500 nM Pc 4, at the light fluence of 200 mJ/cm^2^, induced 16, 26 and 87% cell killing, respectively (Fig. 2A). Treatment of cells with the combination of PDT and dasatinib, each used at LD <30, led to 72% cell killing, which was significantly greater than that of each treatment alone (Fig. 2B). Because both agents are apoptotic inducers (11,12,22–26), the requirement of caspases in cell killing by each agent and the combination was assessed using the pan caspase inhibitor zVAD-fmk. As shown in Fig. 2B, zVAD-fmk substantially inhibited cell killing after dasatinib, but not after either PDT alone or the combination. Overall, the data demonstrate that each agent induces dose-dependent cell killing, the combination enhances cell killing, and that, unlike PDT alone or the combination, dasatinib induces zVAD-fmk-dependent cell killing.

### Dasatinib-induced caspase-3 activation is inhibited by zVAD-fmk. The combination potentiates PDT- or dasatinib-induced activation of caspase-3 in the absence of appearance of other apoptotic markers

**Figure 3.**
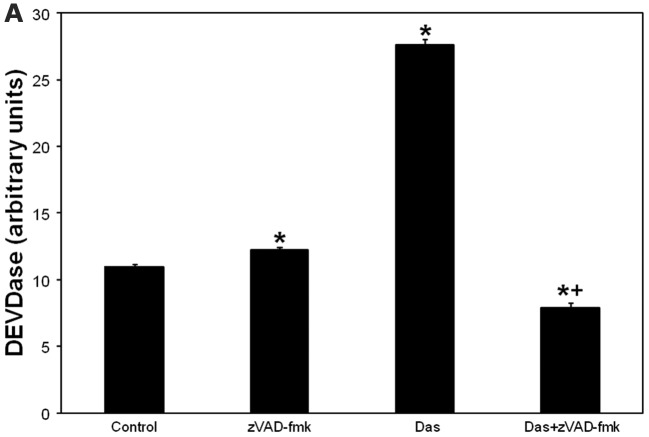

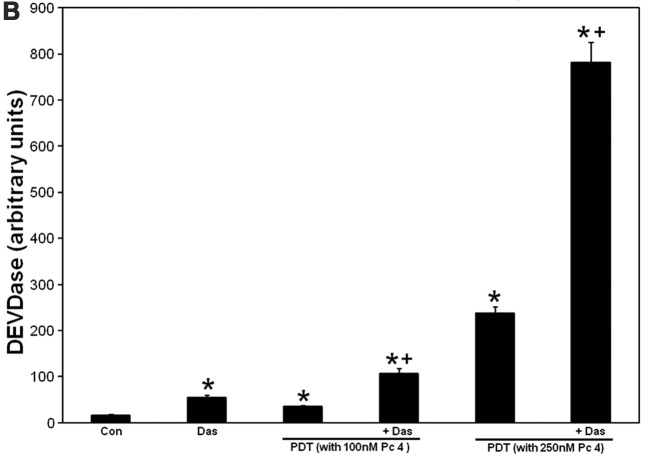

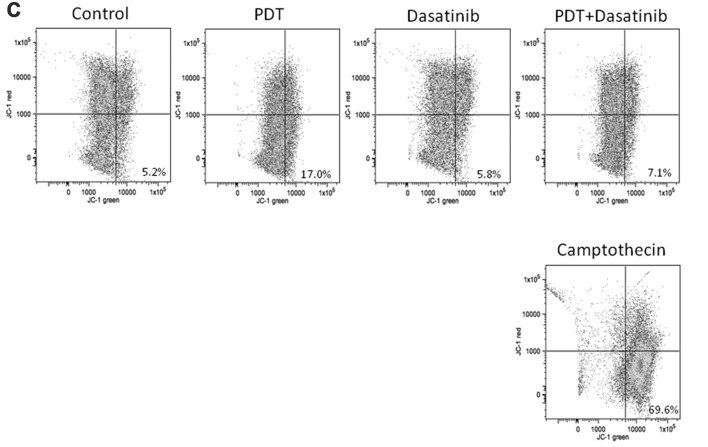

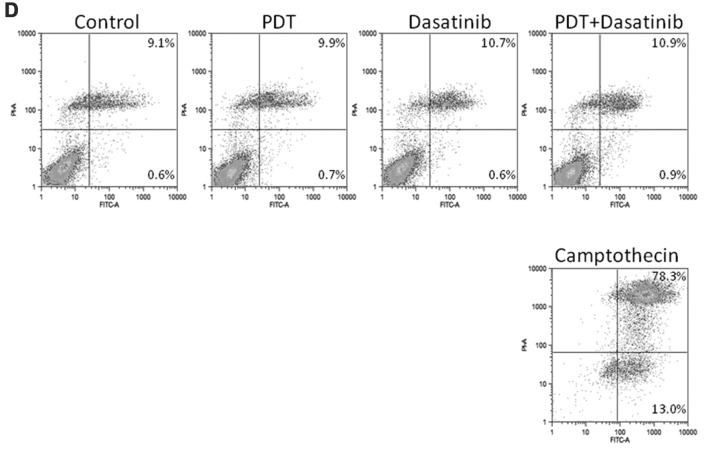
(A) Dasatinib-induced caspase-3 activation is abolished by zVAD-fmk. (B) PDT- or dasatinib-induced caspase-3 activation is potentiated after PDT + dasatinib. (C) PDT-induced mitochondrial depolarization is abolished after the combination. (D) Annexin V^+^ and propidium iodide+ cells remain at control levels after treatments. SCCVII cells were treated with dasatinib (200 nM) for 24 h (A–C) or 72 h (D). For PDT, after overnight incubation with Pc 4 (100 or 250 nM), cells were irradiated with red light (200 mJ/cm^2^) and then incubated for 2 h (B and C) or 48 h (D). For dasatinib + zVAD-fmk, the inhibitor was added 1 h prior to dasatinib (A). For PDT + dasatinib, dasatinib was added to the cells 22 h (B and C) or 24 h prior to irradiation (D). After incubations, cells were collected, lysed and DEVDase assay was carried out to assess caspase-3 activity (A and B). Alternatively, collected cells were processed for flow cytometry using JC-1 or Annexin V/propidium iodide staining for mitochondrial depolarization (C) and apoptosis detection (D), respectively. (C and D) Cells were treated overnight with camptothecin (5 *μ*M). (A and B) The data are shown as the mean ± SEM, n=3–23. The significance (p<0.05) is shown as follows: ^*^treated is different from untreated control; ^+^(PDT + dasatinib) or (dasatinib + zVAD-fmk) is different from individual treatments. Con, untreated control; Das, dasatinib. (C) Percentage of cells with depolarized mitochondria is shown in lower right dot plot. (D) Percentage of Annexin V^+^/propidium iodide^−^ and Annexin V^+^/propidium iodide^+^ cells is shown in lower right and upper right dot plot, respectively.

Because zVAD-fmk inhibits effector caspases, including caspase-3 (27), we verified caspase-3 as a zVAD-fmk target in dasatinib-induced cell death using DEVDase assay. Caspase-3 activation began at 2 h and peaked at 24 h after dasatinib (not shown). As depicted in Fig. 3A, dasatinib induced activation of DEVDase was abolished by zVAD-fmk. The data suggest that zVAD-fmk-sensitive cell killing after dasatinib involves caspase-3.

To further assess induction of apoptosis after treatments, caspase-3 activation, mitochondrial depolarization and the appearance of Annexin V^+^ and propidium iodide+ cells were determined. PDT induced a dose-dependent activation of caspase-3 and the effect was potentiated after PDT + dasatinib (Fig. 3B). PDT alone induced mitochondrial depolarization and the effect was inhibited after the combination (Fig. 3C). Annexin V^+^ and/or propidium iodide+ cells remained at control levels after treatments (Fig. 3D). We validated that SCCVII cells display depolarized mitochondria and undergo apoptosis using camptothecin as a positive control (Fig. 3C and D). The results show that the combined treatment augments PDT or dasatinib-induced caspase-3 activation in the absence of appearance of other apoptotic markers.

### Dasatinib-induced upregulation of mRNA ceramide synthase 1 is enhanced after the combination

**Figure 4.**
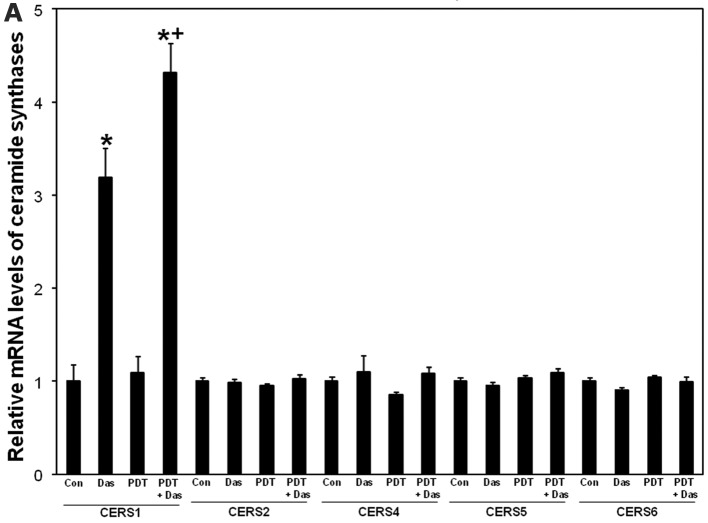

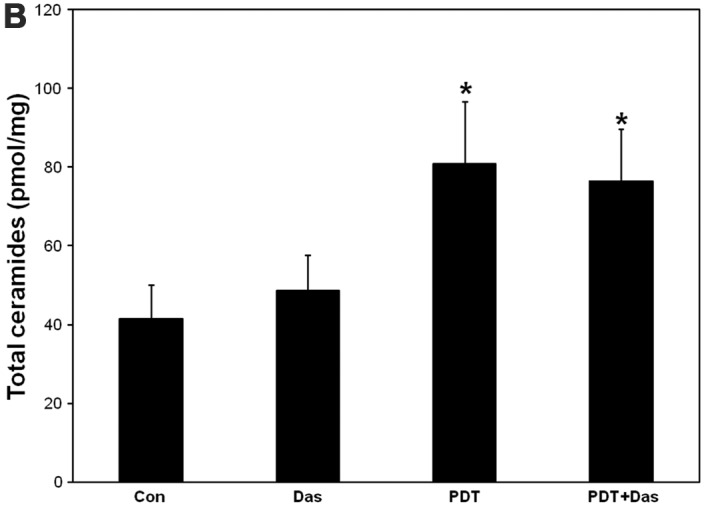

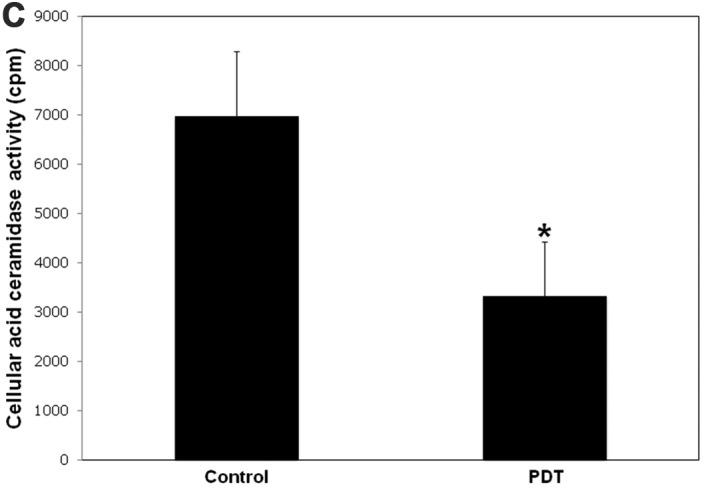
Effect of PDT ± dasatinib on mRNA levels of ceramide synthases (A) or total ceramide levels (B). (C) Acid ceramidase activity is inhibited after PDT. After PDT with Pc 4 [100 nM (A and B)] or 250 nM (C) and the light fluence of 200 mJ/cm^2^, cells were incubated at 37°C for 2 h. (A and B) Cells were treated with dasatinib (200 nM) alone for 24 h. For (PDT + dasatinib), dasatinib was added to the cells 22 h prior to irradiation. After treatments, cells were collected and processed for RT-PCR (A), MS (B) or acid ceramidase assay (C). (A) The data were calculated as: (A) the relative normalized expression of corresponding ceramide synthase mRNAs against the expression of housekeeping gene-encoded proteins RPL37A and HGPRT; (B) pmol/mg protein, and (C) cpm minus background. Control values refer to: (A and B) untreated controls; (C) combined untreated and Pc 4-treated controls. Pc 4 itself had no effect on acid ceramidase activity. All the data are shown as the mean ± SEM, n=4–5. The significance (p<0.05) is shown as follows: ^*^treated is different from control; ^+^(PDT + dasatinib) is different from PDT or dasatinib alone. CERS, ceramide synthase; Con, untreated control; Das, dasatinib.

Dasatinib upregulates expression levels of *ceramide synthase genes 1, 2, 5* and *6* (13). We showed that knockdown of ceramide synthase 1 or 6 leads to apoptotic resistance to PDT (11,12). To test whether ceramide synthases are affected by treatments, mRNA levels of ceramide synthases 1, 2 4, 5 and 6 were measured using RT-PCR. As depicted in Fig. 4A, ceramide synthase 1 mRNA levels were upregulated after dasatinib and the effect was further increased after PDT + dasatinib. PDT alone did not significantly increase ceramide synthase 1 levels. None of the treatments had any effect on mRNA levels of ceramide synthase 2, 4, 5 and 6 (Fig. 4A). The data show that dasatinib-induced upregulation of ceramide synthase 1 mRNA levels is enhanced after the combination.

### Effects of treatments on the sphingolipid profile

To assess whether enhanced upregulation of ceramide synthase 1 mRNA after PDT + dasatinib is associated with increased ceramide production, ceramide levels were measured using MS. Dasatinib alone induced a modest increase in C20:1- and C22-ceramide but had no effect on total ceramide levels (Table I and Fig. 4B). PDT alone increased the levels of all 12 individual ceramides that were measured, as well as total ceramides. PDT-induced increases in ceramide levels were not, for the most part, further changed after the combination, with the exception of attenuated levels of C26- and C26:1-ceramide after PDT + dasatinib compared to individual treatments (Table I and Fig. 4B).

The effects of treatments on other sphingolipids were also measured by MS and are shown in Table I. Unlike dasatinib, and irrespective of the presence of dasatinib, PDT increased the levels of C16-dihydroceramide, an intermediate from the *de novo* sphingolipid biosynthesis pathway. Moreover, unlike dasatinib, and irrespective of the presence of dasatinib, PDT induced an 87% decrease in sphingosine levels. PDT also induced a 62% decrease in S1P levels, and the effect was not changed after the combination. Overall, the data show that in SCCVII cells PDT induced substantial changes in the sphingolipid profile that were not modulated by the addition of dasatinib.

### Acid ceramidase activity is inhibited after PDT

The substantial decrease in sphingosine levels after PDT could result from inhibition of ceramidase, a sphingosine-producing enzyme (Fig. 1). The inhibition of acid ceramidase has been implicated in radiosensitization of prostate cancer cells (28). We measured the activity of acid ceramidase in cell lysates from untreated and PDT-treated cells. As depicted in Fig. 4C, acid ceramidase activity was reduced by 52% post-PDT. Thus, PDT-induced decrease in sphingosine correlated with inhibition of acid ceramidase.

## Discussion

Dasatinib-induced caspase-3 activation and cell killing was zVAD-fmk-dependent. PDT induced caspase-3 activation and the effect was potentiated after the combination. As shown previously (29), PDT-induced caspase-3 activation was abolished by zVAD-fmk. However, PDT- or PDT + dasatinib-induced cell killing was zVAD-fmk-insensitive. zVAD-fmk-insensitivity of PDT is consistent with the findings that caspase-3 is not required for the lethal effect of PDT (30). Incidentally, zVAD-fmk has been reported to upregulate caspase-9 activity in cell death after etoposide (27). We found no activation of caspase-9 after PDT in SCCVII cells (31). Therefore, it is unlikely that zVAD-fmk would have such an effect, especially since the inhibitor was used in our studies at a non-toxic, and comparably, lower concentration than in the etoposide study, i.e., 25 vs. 50 *μ*M, respectively. Overall, the data suggest that, unlike PDT or the combination, dasatinib requires caspase-3 activation for cell killing. Apparently, PDT rather than dasatinib determined zVAD-fmk sensitivity of cell killing after the combination.

Dasatinib-induced upregulation of ceramide synthase 1 mRNA correlated with increased production of C20:1- and C22-ceramide concomitant with activation of caspase-3. This is consistent with the finding that *ceramide synthase 1* gene is upregulated during apoptosis after dasatinib (13). However, there was no correlation between combination-induced enhanced ceramide synthase 1 mRNA upregulation and ceramide production, suggesting that the enzyme might modulate other cellular functions. Ceramide synthase 1 has been associated with sensitization to cisplatin via activation of p38 mitogen-activated protein kinase (MAPK) and concomitant translocation of the enzyme from the endoplasmic reticulum to the Golgi apparatus (32). Activation of p38 MAPK is critical for the antileukemic effects of dasatinib (33). Activation of p38 MAPK pathway has been associated with triggering apoptosis after Pc 4-PDT (34). The potential link between ceramide synthase 1 upregulation, p38 MAPK pathway and sensitization to PDT should be addressed in our future studies.

In HNSCC *in vitro* and *in vivo* models ceramide synthase 1-dependent C18-ceramide production has a proapoptotic role (35) and inhibits xenograft growth (36), respectively. We showed in HNSCC cells that knockdown of CERS1 induced apoptotic resistance to PDT and reduced the levels of total ceramide and several individual ceramides, including C18-ceramide (12). Our findings that the levels of the whole spectrum of ceramides are increased after PDT in the absence of upregulation of ceramide synthase mRNAs imply the involvement of other enzymes. Accordingly, in the present study we have demonstrated that PDT-induced inhibition of acid ceramidase correlates with decrease in sphingosine and increase in ceramide levels, concomitant with activation of caspase-3. Consistent with these findings, upregulation of acid ceramidase confers radioresistance in prostate cancer cells (28). As suggested in the same study, inhibition of acid ceramidase could be a potential target for treatment of cancers with overexpressed acid ceramidase.

The present study shows for the first time enhanced additive killing of SCCVII cells after the combination of PDT and dasatinib and paves the way for testing the combination *in vivo*. This combination has the potential to achieve what is hoped by combination therapy, i.e. maximizing the efficacy of each single anticancer agent while minimizing their systemic toxicity through the delivery of lower drug doses (37). This will have to be validated *in vivo*. Regardless, our novel findings imply the translational potential of the combination for cancer treatment.

## Figures and Tables

**Table I.** Effect of PDT± dasatinib on sphingolipids in SCCVII cells.

Sphingolipid	Untreated	Dasatinib	PDT	PDT + dasatinib
C14-ceramide	13.1±1.4	15.2±1.0	**23.6±1.6^a^**	**21.8±1.4^a^**
C16-ceramide	94.8±11.4	103.9±8.1	**196.5±6.1^a^**	**179.2±20.4^a^**
C18-ceramide	6.8±0.9	8.2±0.5	**21.5±1.6^a^**	**21.5±1.8^a^**
C18:1-ceramide	4.3±0.5	4.4±0.9	**10.5±0.1^a^**	**9.5±0.6^a^**
C20-ceramide	5.8±0.8	7.3±0.7	**15.8±0.6^a^**	**17.6±0.9^a^**
C20:1-ceramide	0.9±0.1	**1.1±0.1^a^**	**2.3±0.2^a^**	**2.5±0.2^a^**
C22-ceramide	32.5±3.8	**44.7±3.5^a^**	**80.4±4.2^a^**	**84.7±4.7^a^**
C22:1-ceramide	10.0±1.1	11.2±1.0	**18.4±0.7^a^**	**17.5±0.7^a^**
C24-ceramide	147.6±15.1	172.3±14.4	**270.6±10.2^a^**	**239.4±12.2^a^**
C24:1-ceramide	171.7±18.8	205.3±15.5	**313.0±14.3^a^**	**307.7±16.6^a^**
C26-ceramide	2.9±0.3	3.4±0.3	**6.7±0.3^a^**	**5.1±0.4^a,b^**
C26:1-ceramide	4.8±0.5	5.5±0.7	**11.7±0.4^a^**	**10.1±0.5^a,b^**
C16-dihydroceramide	10.3±1.5	9.6±0.6	**22.4±0.5^a^**	**19.7±1.1^a^**
Dihydrosphingosine	14.2±2.2	16.9±1.4	17.2±1.3	17.4±0.6
Dihydrosphingosine-1-phosphate	0.4±0.1	0.3±0.1	0.6±0.1	0.5±0.1
Sphingosine	118.8±19.5	150.3±8.7	**15.1±1.0^a^**	**19.6±1.8^a^**
Sphingosine-1-phosphate	1.3±0.1	1.8±0.2	**0.5±0.1^a^**	**0.7±0.1^a^**

Cells were treated with dasatinib (200 nM) for 22 h prior to PDT (100 nM Pc 4 + 200 mJ/cm^2^), and then incubated further for 2 h. Afterwards cells were collected and processed for MS. The data were calculated as pmol/mg protein and are shown as the mean ± SEM (n=3–5).

a Treated is different from untreated control;

b PDT + dasatinib is different from individual treatments (p<0.05).
